# Robotic Percutaneous Coronary Intervention in Real-World Practice: Performance, Complexity, and Learning Curves in Focus

**DOI:** 10.1016/j.jscai.2025.103811

**Published:** 2025-07-23

**Authors:** Ioannis Skalidis, Philippe Garot, Antoinette Neylon, Thomas Hovasse, Mariama Akodad

**Affiliations:** Institut Cardiovasculaire Paris-Sud, Hôpital Jacques Cartier, Ramsay-Santé, Massy, France

The multicenter PRECISION and PRECISION GRX studies represent a significant contribution to the evolving field of robotic-assisted percutaneous coronary intervention (R-PCI).^1^ By systematically comparing first-generation and second-generation robotic platforms across over 1700 procedures, the authors provide robust evidence supporting the feasibility and safety of R-PCI in a real-world setting. Notably, the second-generation CorPath GRX system (Corindus, A Siemens Healthineers Company) demonstrated comparable overall clinical and technical success to its predecessor, while enabling higher success in more complex anatomies, including lesions with moderate/severe calcification and bifurcation lesions.

A particularly relevant advancement is the robotic guide catheter control in the GRX system, which likely contributed to reduced manual conversions and improved outcomes in challenging lesion subsets. The lower contrast volume observed despite longer procedural times in PRECISION GRX raises interesting questions regarding the efficiency gains from enhanced robotic control and visualization capabilities. Further granularity on contrast-saving mechanisms, possibly linked to less frequent or more targeted angiographic imaging, would be valuable.

Additionally, while manual intervention remained necessary in approximately 11% of cases in both cohorts, distinguishing between transient and complete conversions—especially in the context of hybrid CTO strategies—highlights the operational flexibility required in current R-PCI practice. It would be insightful to explore whether specific procedural planning algorithms or lesion scoring tools could prospectively stratify R-PCI suitability, particularly to avoid unplanned conversions.

Another important consideration is the operator learning curve. While multicenter design and high overall success rates are reassuring, the studies do not stratify outcomes by operator experience or volume. Given the technical specificity of R-PCI and potential differences in tactile feedback, it would be valuable to assess whether a threshold number of cases is required to reach procedural efficiency and optimize outcomes, particularly in complex lesion subsets. Standardized training pathways and proficiency benchmarks could be instrumental for broader adoption and equitable outcomes across institutions.

Looking forward, these findings by Mahmud et al^1^ underscore the potential for robotic platforms to expand beyond procedural assistance to teleoperation. However, broader adoption will depend on addressing current limitations, such as the need for dual robotic drives for bifurcation stenting and improved device exchange automation. These results prompt further investigation into how technological refinements and operator training pathways might synergistically elevate the role of R-PCI.

## References

[1] Mahmud E., Madder R.D., Wohns D.H. (2025). Robotic-assisted percutaneous coronary intervention: Final results of the PRECISION and PRECISION GRX studies. J Soc Cardiovasc Angiogr Interv.

